# Real-time cancer diagnosis of breast cancer using fluorescence lifetime endoscopy based on the pH

**DOI:** 10.1038/s41598-021-96531-0

**Published:** 2021-08-19

**Authors:** Jooran Lee, Byungyeon Kim, Byungjun Park, Youngjae Won, Sang-Yeob Kim, Seungrag Lee

**Affiliations:** 1 Medical Device Development Center, Osong Medical Innovation Foundation, Cheongju, Chungbuk 28160 South Korea; 2 Intek-Medi, 123, Osongsaengmyeong-ro, Osong-eup, Heungdeok-gu, Cheongju-si, Chungcheongbuk-do South Korea; 3 Asan Institute for Life Sciences, Asan Medical Center, University of Ulsan College of Medicine, 88 Olympic-ro, 43-gil, Songpa-gu, Seoul, 138-736 South Korea

**Keywords:** Cancer, Systems biology, Optics and photonics

## Abstract

A biopsy is often performed for the diagnosis of cancer during a surgical operation. In addition, pathological biopsy is required to discriminate the margin between cancer tissues and normal tissues in surgical specimens. In this study, we presented a novel method for discriminating between tumor and normal tissues using fluorescence lifetime endoscopy (FLE). We demonstrated the relationship between the fluorescence lifetime and pH in fluorescein using the proposed fluorescence lifetime measurement system. We also showed that cancer could be diagnosed based on this relationship by assessing differences in pH based fluorescence lifetime between cancer and normal tissues using two different types of tumor such as breast tumors (MDA-MB-361) and skin tumors (A375), where cancer tissues have ranged in pH from 4.5 to 7.0 and normal tissues have ranged in pH from 7.0 to 7.4. To support this approach, we performed hematoxylin and eosin (H&E) staining test of normal and cancer tissues within a certain area. From these results, we showed the ability to diagnose a cancer using FLE technique, which were consistent with the diagnosis of a cancer with H&E staining test. In summary, the proposed pH-based FLE technique could provide a real time, in vivo, and in-situ clinical diagnostic method for the cancer surgical and could be presented as an alternative to biopsy procedures.

## Introduction

Breast cancer is one of the most frequent tumors affecting women worldwide and is considered the second leading cause of death in women^1^. Accordingly, increases have been reported in the diagnosis and surgical treatment of breast cancer. According to clinical research on breast cancer, the margin of tumor resection is highly important in diagnosis and treatment because breast cancer resection can affect women’s appearance and quality of life. To facilitate the diagnosis of cancer during a surgical operation, biopsy is generally performed by a surgeon to determine the presence of tumor tissues by using hematoxylin and eosin (H&E) staining under an optical microscopy by a pathologist. However, this biopsy process which involve resection and H&E staining examination can be time consuming for a surgical operation and can be challenging for the determination of an accurate tumor margin.

In order to access the evaluation of a cancer in vivo without a biopsy, optical techniques over the past 10 years has been development such as Raman spectroscopy^2–4^. These techniques have utilized Raman scattering that allows the analysis of a bio-chemical compositions in cell level. Recently, hand-held Raman spectroscopy probe system have been described in diagnosis of brain cancer^5^. Although these techniques are a promising diagnostic tool for cancer screening and the intraoperative surgical guidance, they require expensive lasers, detector, and optical components such as filters. Due to the weak signal of Raman scattering, the scattered signal is required to be strongly enhanced using metal nanoparticles such as SERS (Surface enhanced Raman spectroscopy). However, most of metal nanoparticles cannot be used for clinical applications due to their safety with the toxicity^6^.

The fluorescence-based methods using fluorescence dyes have focused on the intensity of fluorescence emitted by a contrast agent that can bind to specific targets associated with cancer or accumulate nonspecifically through leaky blood vessels. Although numerous fluorophore studies have been attempted^7–9^, few fluorescent dyes have been approved by Food and Drug Administration (FDA) for clinical use such as fluorescein^10^, methylene blue^11^, indocyanine green^12^, and 5-aminolevulinic acid^13^. In particular, fluorescein have been widely used to diagnose a cancer in gastroenterology with confocal laser endoscopy system that allows the minimally invasive discrimination of an abnormal lesion on a tissue surface in vivo with high resolution cell imaging^14,15^. These clinical approaches have enabled surgeons to immediately determine the histopathological evaluation and the diagnosis of a tumor without biopsy during a surgical operation.

One representative medical device currently available for commercial use is the Cellvizio confocal endomicroscopy (Mauna Kea Technologies, France). This endomicroscopy provides a microscopic cell imaging based on the fluorescence intensity of fluorescein which is nonspecifically accumulated through leaky blood vessels and can allow the diagnosis of a cancer based on morphological patterns of normal tissues and cancer tissues. On the other hand, physician or specialist have to determine the presence or absence of a lesion by his subjective decision since it can only provide a qualitative fluorescence intensity information.

In this fluorescence intensity imaging method, the concentration quenching occurs as the concentration of the fluorescing molecule increases in a sample. It decreases fluorescence intensity in highly concentrated solution. Autofluorescence can reduce image quality in the fluorescence intensity imaging in vivo^16^. To minimize these problems, laser power, a concentration of fluorophore, and the detected wavelength through an optical filter in confocal endomicroscopy based on the fluorescence intensity with a contrast agency. However, fluorescence lifetime measurement can provide the quantitative information as an indicator to monitor the change of pH, ion concentration and refractive index regardless of the fluorescence intensity with a laser power and a concentration of fluorophore. Taken together, fluorescence lifetime imaging can be a powerful and useful tool to facilitate the diagnosis of tumor tissues for clinical applications rather than fluorescence intensity imaging^17,18^.

The fluorescence lifetime of a molecule is the average time that the molecule spends in the excited state after absorbing extremely short-pulsed laser energy^19^. Molecular fluorescence lifetime is not dependent on laser power, concentration of the molecule, or photobleaching, and it can effectively monitor biochemical information in areas of interest in a cell or tissue because fluorescence lifetime is not sensitive to fluorescence intensity, but only to the energy transfer between the fluorophore and its environment^20^. As an alternative, Kumar et al.^21^ reported the fluorescence lifetime-based contrast enhancement of indocyanine green-labeled tumors. This fluorescence lifetime-based system could provide more than 98% sensitivity and specificity and a tenfold reduction in error rates compared with intensity-based detection. Nevertheless, this technique still has problems; for example, ICG should not remain in the normal tissue for contrast enhancement, it takes a few hours to a day after injection.

We have recently proposed a new optical method that can be used for immediate diagnosis after dropping the fluorescence probe based on differences in the pH of cancer tissues and normal tissues^22^. In our previous works, we already described a real-time fluorescence lifetime technique, called analog mean-delay (AMD), for observing dynamic biological reactions, making medical diagnoses, and performing real-time industrial inspections^20,23^.

In this study, we present an endoscopic fluorescence lifetime imaging technique to provide the ability to discriminate health and tumor tissues in two different types of mouse model with a breast cancer and a skin cancer. To verify the change of pH-related fluorescence lifetime, we showed the relation between pH and fluorescence lifetime at three different concentrations of fluorescein with the proposed system, compared to time-correlated signal photon counting (TCSPC) based fluorescence lifetime measurement system. We demonstrated the feasibility of in real time, in vivo, and in-situ cancer diagnosis by visualizing and analyzing the fluorescence lifetime information of tissues over a certain area without the resection of tissues for breast cancer and skin cancer(N = 20 mice). We also confirmed the results of pH-related fluorescence lifetime corresponded to the results of the H&E staining test for normal tissues and tumor tissues.

## Results

### Real-time AMD-FLE endoscope system

**Figure 1:**
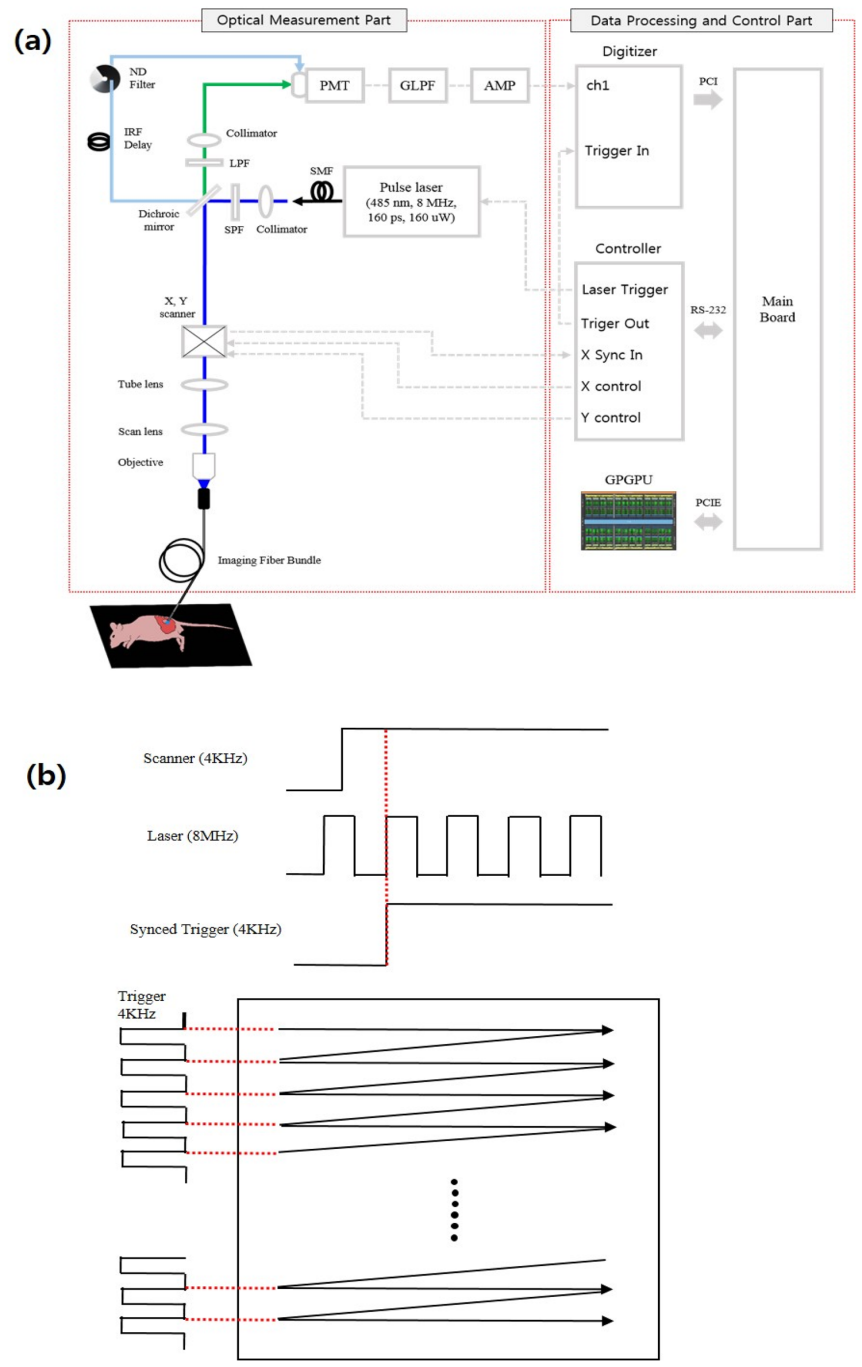
(**a**) Schematic illustration of the experimental set-up. (**b**) Signal chronogram of trigger synchronization.

The basic configuration of the proposed system is the combination of the real time analog mean delay (AMD)-fluorescence lifetime microscopy (FLM) and optical endoscopy. We explained the optical setup of AMD-FLM and the real time fluorescence lifetime measurement using high-speed parallel signal processing based on GPU in Material and Methods. In the study, we illustrated a real-time fluorescence lifetime imaging system was coupled with an endoscopic method which could be applicable to discriminate a cancer without a biopsy during a surgical operation as shown in Fig. 1a. In order to implement endoscope system, we used objective lens (20 × /0.45, MPlanFL; Olympus) as a combination part between the scan lens and the imaging fiber bundle system manufactured by Mauna kea technologies, where the imaging fiber bundle system consisted of the fiber bundle and imaging lens with field of view (240 µm by 240 µm), lateral resolution (≈1 µm), and working distance (55 µm ~ 65 µm). Imaging fiber bundle used for endoscope system had a diameter of about 2.6 mm, a length of 3 m, and 30,000 core pixels with a diameter of 3 µm. The imaging bundle was situated at the focal position of the objective lens. The laser beam passed to the imaging fiber bundle system through the scan lens and excited to the tissue in Fig. 1a. After the reflected emission beam was gathered by PMT connected to digitizer, we could obtain 2D fluorescence lifetime information of tissues over a certain area at the speed of 2 images per second. The proposed endoscopic system can be utilized in gastroenterology by inserting the imaging fiber bundle into a biopsy channel of a commercial endoscope and in surgery itself for the needs of a clinical applications.

### Changes in the fluorescence lifetime of the fluorescein according to pH

**Figure 2:**
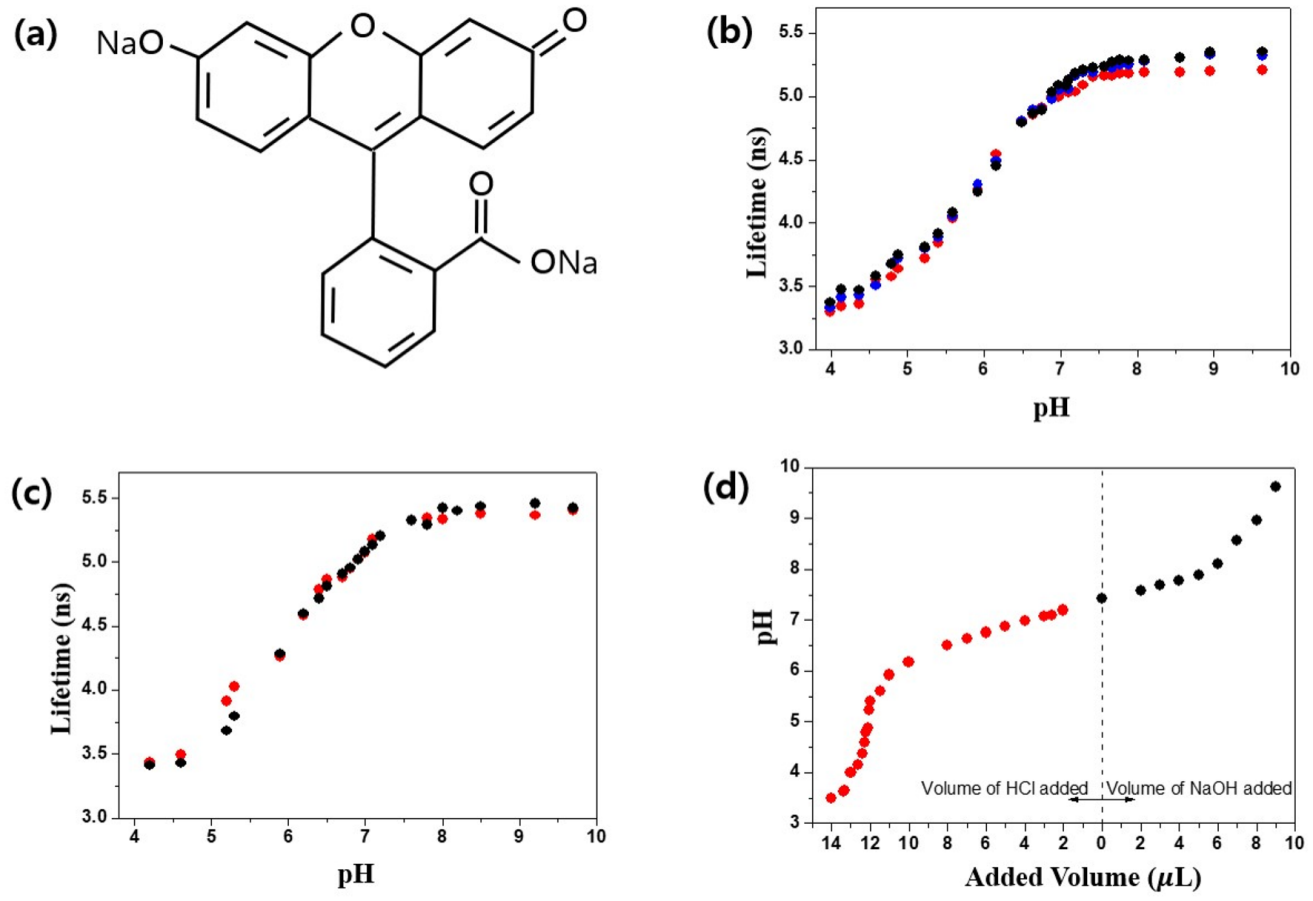
(**a**) The structures of pH indicators used in this study. (**b**) Fluorescence lifetime versus pH of sodium fluorescein excited by 485 nm laser with FLE at three different concentrations with 25 µg/mL (Red dots), 50 µg/mL (Blue dots), and 75 µg/mL (Black dots) between pH 3.9 and pH 9.6. (**c**) Fluorescence lifetime versus pH of 25 µg/mL sodium fluorescein by 485 nm with FLE (Red dots) or TCSPC (Black dots). (**d**) Change in pH of PBS by the addition of 20% HCl (Red dots) or NaOH (Black dots) solution.

The fluorescence lifetime of sodium fluorescein depends on pH but not on temperature (22 to 37 degree) or molecule concentrations at the clinical values found in blood vessels and tissues^24,25^. The change in fluorescence lifetime with pH is related to the structure of fluorescein. As the pH decreases, protons (H +) are added and the structure changes^26^. Fluorescein absorbs light of 490 nm wavelength and emits a green light around 515 nm. When pH > 8, absorption and emission are greatest. Due to the equilibrium between phenol fragments and carboxyl functions and lactones, fluorescein’s ionic charge and chemical structure evolve with the ambient pH, causing fluctuations in its photophysical properties. At 490 nm excitation, the fluorescence quantum yield is very high under basic conditions (pH > 8), but acidification of the solution gradually leads to fluorescence extinction. This is because fluorescein in its anionic form transitions to anionic equilibrium, resulting in lower absorbance associated with blue shift. In other words, as the pH decreases, the quantum yield decreases and the fluorescence lifetime shortened according to the following equation: *Φ*_*f* =_ k_r_ × τ_f_ where *Φ*_*f*_ is the fluorescence emission quantum yield, τ_f_ is the fluorescence lifetime and k_r_ is the constant. Figure 2a shows the structure of sodium fluorescein as a pH indicator used in this study.

In order to demonstrate the relation between fluorescence lifetime and pH using the proposed AMD-FLE system in three different concentration conditions of sodium fluorescein (25, 50, and 75 µg/mL). We followed the previous results reported by Hammer et al.^24^. We used sodium fluorescein as a pH indicator to measure the change of a fluorescence lifetime for pH within a range from 3.9 to 9.6. Additionally, we measured the fluorescence lifetime of sodium fluorescein with different concentration of 25, 50, and 75 µg/mL to confirm that fluorescence lifetime was not dependent on concentration but pH in fluorescein. We used 485 nm pulse laser with a width of 30 ps and a repetition rate of 8 MHz as an excitation optical source to fluorescein (Fig. 2b). These results revealed there existed the relation between fluorescence lifetime and pH in fluorescein. It can be clearly seen that lifetime increased steadily to 5.4 ns as pH increased from 3.9 to 7.4 and then lifetime remained stable at approximately 7.8. These results indicated that the proposed measurement technique with fluorescence lifetime imaging can distinguish between cancer tissues and normal tissues since cancer tissues show a decreased pH, compared to normal tissues. We also found there was a similar trend in lifetime versus pH regardless of concentration of fluorescein. These results were in good correspondence to the previous study^24^. As illustrated in Fig. 2c, we also measured fluorescence lifetime versus pH in fluorescein using a commonly used time-correlated single photon counting (TCSPC) equipment produced by PicoQuant (FluoTime 300, www.picoquant.com) to prove our results with AMD-FLE system. Our findings showed the results of our proposed fluorescence lifetime measurement correlated well with that of the commercial product, which illustrated our proposed system showed a good performance with an accuracy for the measurement of a fluorescence lifetime. Figure 2d shows the change in pH of PBS by the addition of 20% HCl or NaOH in sodium fluorescein solution. We made fluorescein solutions with a different pH and then measured the pH in fluorescein solution using pH measurement device (LAQUA 9615S; HORIBA). The titration of either a strong acid with a strong base or a strong base with a strong acid produces an S-shaped curve^27^. Fluorescence lifetime of Fig. 2 were calculated by averaging 10,000 samples over an area of 100 by 100 pixels in 2D fluorescence lifetime image. We utilized its mean value as a diagnostic indicator for normal and cancer tissues, where the sensitivity of pH in our system was approximately 0.06 using a least-square line fit within a range from pH 6.5 to pH 7.6 from the sensitivity (≈30 ps) of the fluorescence lifetime measurement referred as our previous study^28^.

### Cancer discrimination based on pH and fluorescence lifetime

To determine whether real-time cancer diagnosis is possible, fluorescein (50 μg/mL) was dropped on the surface of tumor and normal tissues in live mouse and then the surface was washed with PBS at three times. Finally, we measured the fluorescence lifetime of normal and tumor tissues using the proposed system.

**Figure 3:**
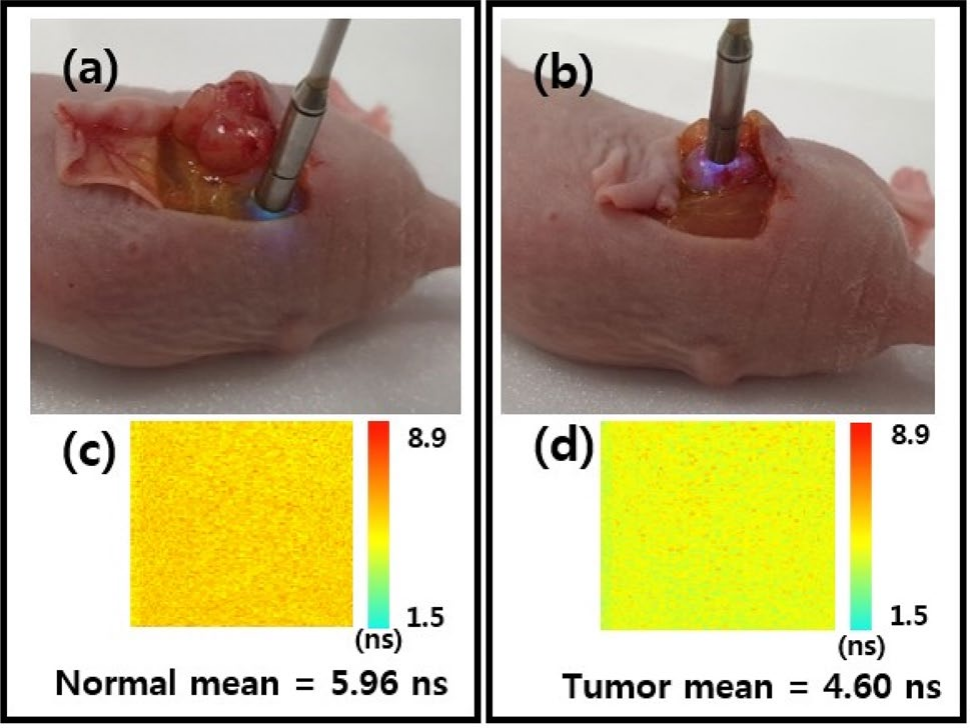
Demonstration of fluorescence lifetime distinction between tumor and normal tissues in mice. (**a**,**b**) The top row shows the fluorescence lifetime measurement images for normal and tumor tissues using the proposed system in mice with subcutaneous MDA-MB-361 breast tumors. (**c**,**d**) Fluorescence lifetime images over 100 by 100 pixels and its mean value in normal tissue of (**a**) and in tumor tissue of (**b**). The color bar represents the fluorescence lifetime in nanosecond.

As shown in Fig. 3c, the mean value of the fluorescence lifetime was 5.96 ns in normal tissue and its pseudo color seemed dark yellow. In contrast, the mean value of the fluorescence lifetime was 4.60 ns in tumor tissue and its pseudo color seem light green in Fig. 3d. These results reported that the fluorescence lifetime of the tumor tissue was lower than that of the normal tissue in mice with breast tumor. Our findings showed that the difference in fluorescence lifetime by the effect of pH in the cancer tissue, compared to the normal tissue. The mean value of the fluorescence lifetime was averaged for 10,000 pixels over an area of 100 µm by 100 µm.

**Figure 4:**
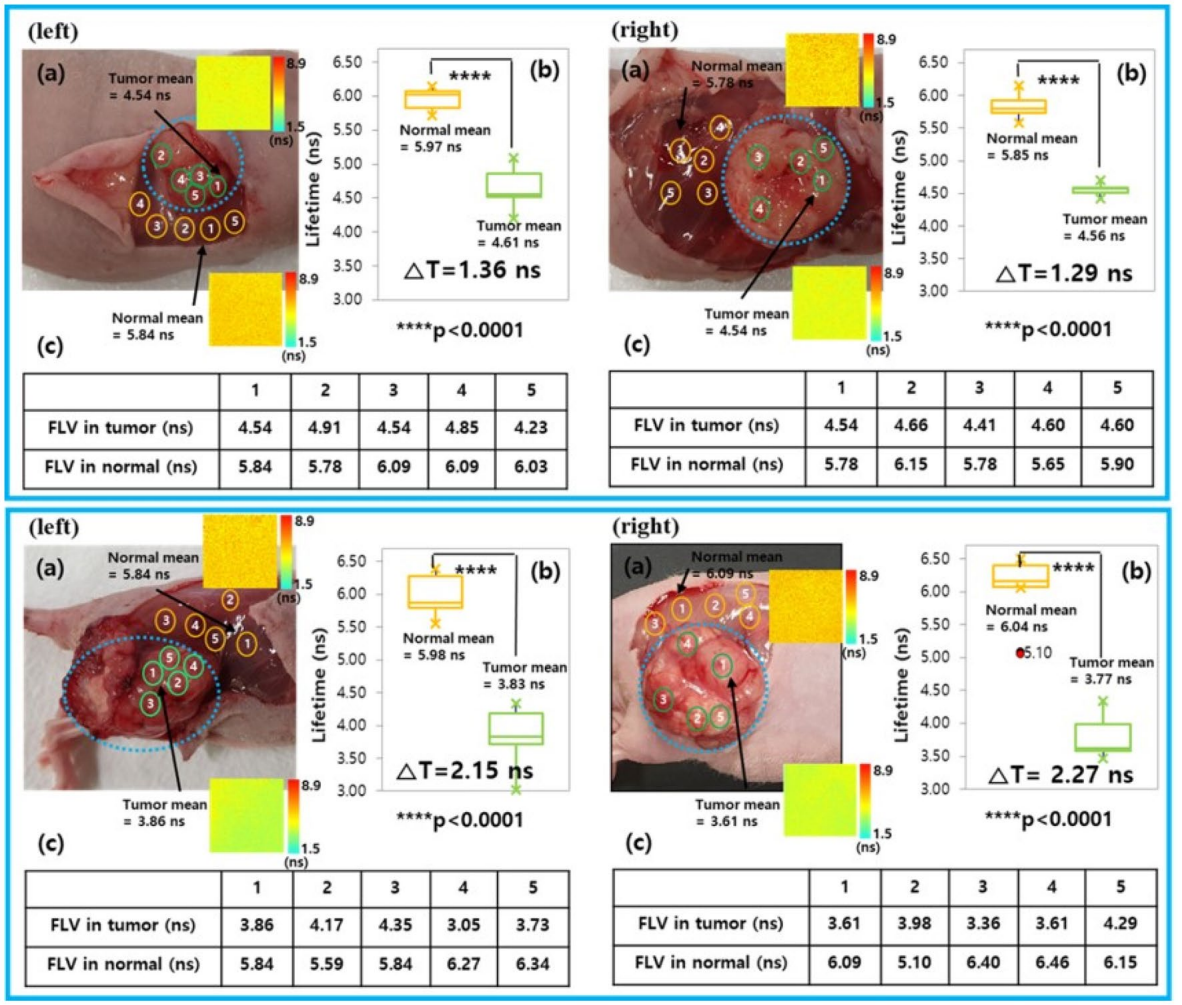
(**a**) Camera images showing the boundary between the cancer tissue and normal area and the point at which the fluorescence lifetime was measured in mice. (**b**) Distribution of fluorescence lifetimes within the tumor and normal tissues. (**c**) Fluorescence lifetime value for each point. The left and right panels represent the fluorescence lifetimes of different tumor sizes; the top and bottom represent the fluorescence lifetimes for MDA-MB-361 breast cancer and A375 skin cancer samples, respectively. (The color bar represents the fluorescence lifetime in nanosecond) (△T means the difference in fluorescence lifetime between the normal mean and tumor mean) (**** *P* < 0.0001, normal tissues versus tumor tissues).

In order to confirm the feasibility to discriminate the boundary between a normal region and a tumor region, we randomly selected five points in their regions for mice with a breast cancer and mice with a skin cancer and fluorescence lifetime was measured 10 times at each point. Fluorescence lifetime value (FLV) was averaged for mean values of fluorescence lifetime measured 10 times at each point. Finally, we obtained the normal mean and the tumor mean by averaging 5 fluorescence lifetime values for 5 points, where the area of the point corresponded to 100 µm by 100 µm. As shown in Fig. 4b,c illustrated the comparison of the fluorescence lifetime distribution and FLV for normal mean and tumor mean, respectively. The FLV ranged from 5.78 to 6.09 ns and its mean was 5.97 ns for normal tissues of breast cancer mice as shown in the top (row)-left (side) of Fig. 4. The FLV ranged from 4.23 to 4.91 ns and its mean was 4.61 ns for tumor tissues of breast cancer mice. From these results, we found the difference in fluorescence lifetime was approximately 1.36 ns.

To determine whether the diagnosis could be made according to the size or type of tumor, tumors were categorized as measuring less than 1 cm (Fig. 4, left) or greater than 1.5 cm (Fig. 4, right) in diameter. A375 skin cancer tumors (Fig. 4, bottom) were also measured several times in the same way described for breast cancer. There were only very minor changes depending on tumor size, and the results confirmed that the difference in the fluorescence lifetime between normal tissues and breast cancer tissues was approximately 1300 ps, whereas that between normal tissues and skin cancer tissues was approximately 2200 ps. These results confirmed that cancer tissues could be distinguished from normal tissues in real-time using the proposed endoscopic fluorescence lifetime based on pH, enabling diagnosis of a cancer without resection of tissues.

**Figure 5:**
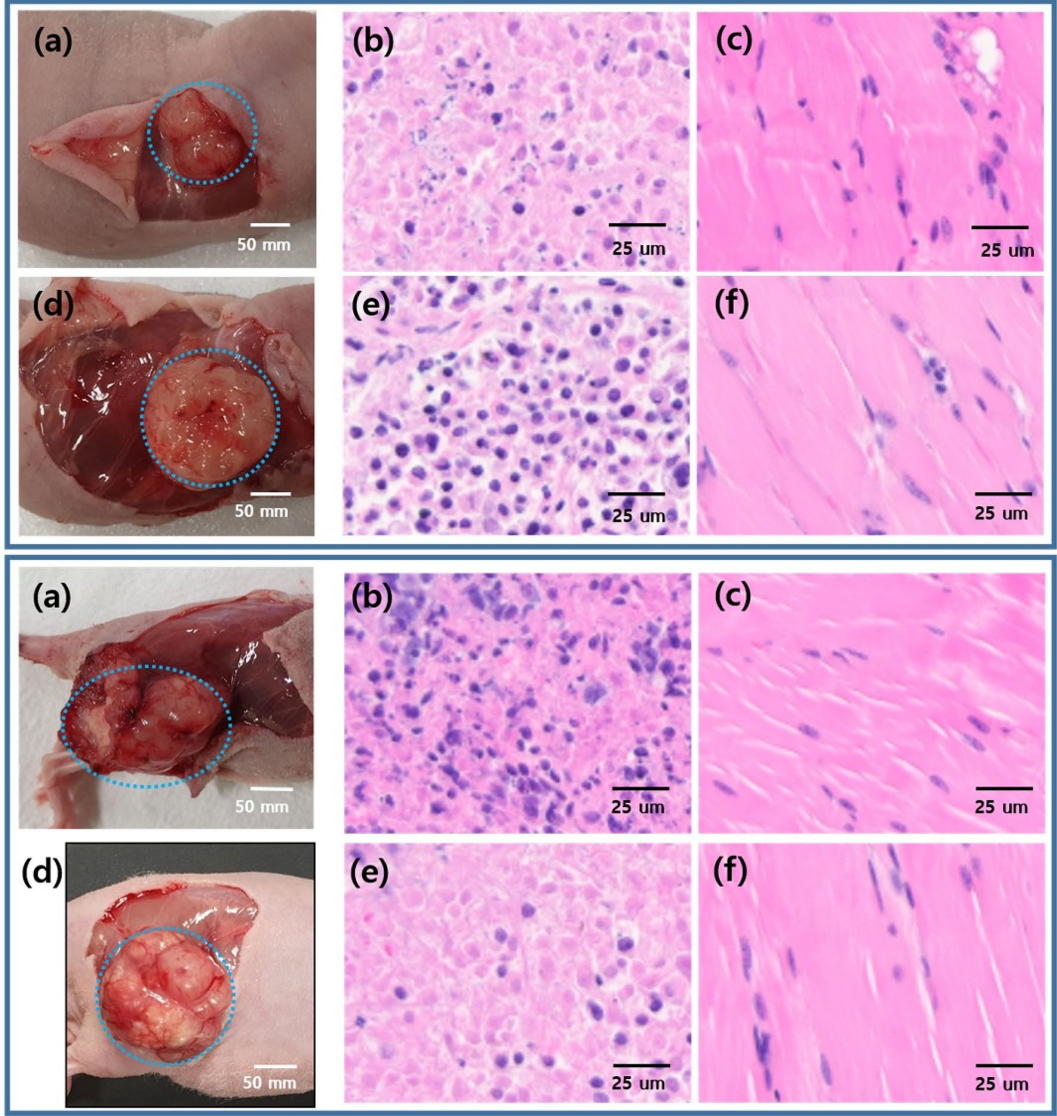
(**a**) Images of the breast cancer model (Top: MDA-MB-361 cell line) and skin cancer model (Bottom: A375 cell line). Hematoxylin and eosin (H&E) staining results of tissues of the normal group (**b**) and tumor group (**c**). Compared with tissues in the normal group, those in the tumor group showed cell and nuclear damage.

Next, we compared our results with results obtained from H&E staining. This technique was used to confirm the presence of tumor tissues within the tissue regions evaluated. Nuclei were stained purple, and cytoplasm was stained pink. Cancer cells were larger in size than normal cells, and their morphology was not uniform. Additionally, we observed giant nuclei, dual nuclei, multiple nuclei, or irregular nuclei in cancer cells, and the ratio of nucleus to cytoplasm in cancer cells was different from that of normal cells (Fig. 5). H&E staining also showed that the nucleoli of MDA-MB-361 and A375 cell derived tumors are prominent, compared to normal tissues. These findings confirmed that the results obtained using our method of analysis were consistent with those obtained from conventional H&E staining.

In order to elucidate difference in fluorescence lifetime for normal and tumor tissues displayed in Fig. 4, we evaluated it between normal tissues (N = 20) and tumor tissues with MDA-MB-361 breast cancer (N = 10) and A375 skin cancer (N = 10) using ANOVA statistical analysis by PRISM 9.1.0.221 (GraphPad Software Inc., https://www.graphpad.com/scientific-software/prism/). Variance in p-value between normal tissues and breast tumor tissues displayed in Fig. 5 clearly verified the difference between two fluorescence lifetime (P < 0.0001), where the averaged lifetime of normal tissues was 5.96 ns and the averaged lifetime of breast tumor tissues was 4.59 ns. The fluorescence lifetime of the two tissues between normal and skin tumor tissues also showed a significant difference in P-value (P < 0.0001), where the averaged lifetime of skin tumor tissues was 3.80 ns.

## Discussion

Breast cancer is one of the most common invasive cancer in women and a fatal disease in which cancer cell forms in the tissues of the breast. In the paper, we have attempted to verify the feasibility of in-situ diagnosis using the fluorescence lifetime imaging technique in breast cancer without the resection of tissues.

Fluorescence lifetime imaging techniques have exploited the lifetime characteristics of fluorescence caused by the change of molecular conformation in cells. Numerous studies have been attempted to apply the intraoperative fluorescence lifetime techniques to cancer diagnosis for surgery^29–35^. The Fruhwirth’s group presented an endoscopic fluorescence lifetime imaging system based on time-correlated single photon counting (TCSPC) with a live cell^31^. The Wang’s group described intraoperative margin assessment in TCSPC fluorescence lifetime microscopy for lung cancer^32^. This limited the assessment of a cancer in real time due to the acquisition time of single photon emitted by a tissue. However, a real-time fiber-based fluorescence lifetime imaging application using TCSPC has been recently proposed by Lagarto’s group^33^. This method showed the real time fluorescence lifetime imaging technique based on TCSPC by single-point measurement method with synchronous external illumination. Marcu’s group have proposed the new approach of the real time fluorescence lifetime imaging by using the pulse sampling technique in terms of the high speed acquisition of a fluorescence signal, which required high speed sampling rate digitizer over 12.5 GS/s^34^. Wide field fluorescence lifetime imaging (FLIM) approaches using time-of-flight (ToF) camera were proposed by The Erkkilä's group^35^. However, this could be only applicable to discriminate normal tissues from tumor tissues in the resected tissues. To improve the signal processing speed associated with the acquisition time of single photon counting, Kim’s group proposed a new fluorescence lifetime imaging system (FLIS) based on AMD (analog-mean delay) in 2009^36^. We developed the high-speed AMD-FLIS using GPU to visualize the fluorescence lifetime image in real time^20^. The fluorescence lifetime imaging system with AMD could help the mechanism identification since it could observe and analyze the biomedical phenomena such as pH, ion concentration, or intracellular environment occurred within 1 s^23^.

In this study, we proposed a new cancer diagnostic optical measurement method that allows for immediate diagnosis after dropping a fluorescent probe on the surface of tissue as a non-invasively manner without the resection of tissues; this method is based on the detection of differences in pH values between cancer and normal tissues using real-time fluorescence lifetime technology. The pH of normal tissues is generally neutral (7.0–7.4), whereas the regions around cancer tissues are acidified owing to increased lactic acid content from aerobic metabolic processes of cancer cells, resulting in a decrease in pH^37–39^. However, in benign tumors, the pH value similar to that of normal tissues is hardly distinguishable. Also, inflammation caused by infection may be slightly acidic in the microenvironment. However, infectious nodules can be weakly acidic, pH-neutral or weakly alkaline, but because they are not strongly acidic, cancer and nodules can be distinguished using pH^40,41^.

According to our method, which exploited FDA-approved fluorescein, the fluorescence lifetime values of cancer and normal tissues differed due to differences in pH values; normal tissues presented higher pH values than cancer tissues, for both breast and skin cancers. In addition, no significant differences in fluorescence lifetimes were observed according to tumor size in mice. This may be related to the use of an epidermal xenograft model, which generally yields tumors with similar sizes according to the initial cancer type. However, in clinical trials, as the size of a tumor increases, the depth changes and the stage of cancer progresses, resulting in differences in pH values^42,43^. The fluorescence lifetime will vary according to differences in pH values of cancer tissues. Thus, the progression of cancer (based on changes in size) could be predicted by measuring the fluorescence lifetime using our system.

Notably, we observed that different types of cancer showed variations in fluorescence lifetime values. For example, the differences between normal tissues and tumors tissues were 1300 and 2200 ps for breast cancer and skin cancer, respectively. It has been reported that pH varies depending on the tissues and organs of the body^39,44,45^. For example, the skin maintains a weak acidity of about 5.5 to protect the body from external infections and the stomach needs to maintain pH 2 in order to digest food. Additional studies using tissues from various types cancer are required. Such findings are expected to facilitate cancer diagnosis in real-time.

Finally, we confirmed the results using conventional H&E staining^46–49^. The results showed that our method yielded results similar to those of H&E staining. Therefore, the proposed pH based FLE approach may be a promising method for real-time cancer diagnosis and treatment in the operating room without tissue resection.

**Figure 6:**
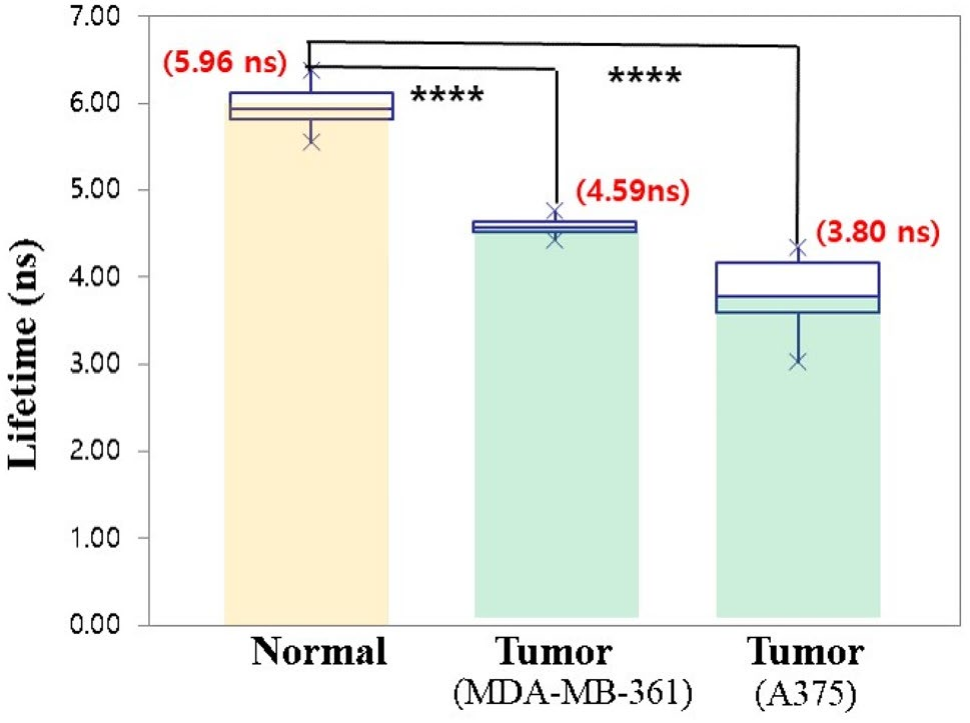
Difference in fluorescence lifetime between the normal tissues and two tumor tissues. (**** P < 0.0001) (Number of normal tissues = 20, Number of breast tumor tissues = 10, Number of skin tumor tissues = 10).

In this study, we verified a relationship between fluorescence lifetime and pH in sodium fluorescein and pH-related fluorescence lifetime imaging technique for cancer diagnosis. Variances of p-value clearly demonstrated a distinct discrimination between normal tissues and cancer tissues in Figs. 4 and 6 (*p* < 0.0001). We provided the mean value of 2D fluorescence lifetime information within 100 by 100 pixels as a diagnosis indicator for normal and cancer tissues.

Based on these finding, we could assume that pH caused difference in fluorescence lifetime in tissues and cancer tissues was lower pH value compared to healthy tissues. I believe that the pseudo color difference in fluorescence lifetime between normal and cancer tissues could be an indicator factor for pH-related cancer discrimination. In addition, although the commercial confocal endomicroscopy (Company: Mauna Kea Technologies, Model: Cellvizio) with fluorescein dye have been extensively utilized to qualitatively determine abnormal lesion or a cancer based on the morphological cell imaging, I suggest that our system can provide the quantitative information of lifetime and it can be advanced optical imaging technique to provide the biochemical properties with pH, ion concentration, or FRET.

Our findings are of great importance for the assessment of cancer with in vivo fluorescence lifetime endoscopy system in intraoperative laparoscopic surgery applications. Nevertheless, we used imaging fiber bundle combined with fluorescence lifetime imaging system based on confocal optical system. Following the principle of confocal imaging with high N. A. objective, our system limited the penetration depth of about 60 µm close to the surface of a tissue. It was sufficient to measure the fluorescence lifetime information of tissues since the emitted fluorescence signal was gathered by contacting the imaging fiber bundle to the surface of tissue as shown in Fig. 3. There could be no effect to determine a cancer on the surface of breast and skin samples transversely as shown in Fig. 5. In the study, we claimed the cancer diagnosis of pH-related fluorescence lifetime by using in vitro pH measurement for sodium fluorescein. Despite promising approach for cancer diagnosis with in vivo pH-related optical measurement in laparoscopic surgery without a biopsy, in vivo pH measurement in tissues as optical biopsy would be needed for clinical applications. In addition, we suggested the cancer diagnosis technique based on fluorescence lifetime for topical applications which might limit a tissue absorption and penetration of the fluorophore. The used fluorescein has been approved for clinical uses based on intravenous administration. To succeed our present technique as a practical medical device, two kinds of issues with in vivo pH measurement and intravenous administration for fluorescein should be discussed and solved by comparing in vivo pH measurement between the proposed system and a commercial pH measurement with intraoperative intravenous fluorescence injection in further studies.

In summary, the presented study has demonstrated the real-time pH-related fluorescence lifetime imaging system can be a practical tool to determine a cancer or abnormal lesion associated with pH by showing difference in fluorescence lifetime between normal and cancer tissues. We believe that the proposed fluorescence lifetime endoscopy, with the help of pH, can be utilized to study the biochemical and physical properties under stage of cancer.

## Materials and methods

### Setup for real-time AMD-FLE

A schematic diagram of the real-time confocal AMD-FLE method is shown in Fig. 1a. Except for the instrument response function (IRF) referencing technique, the optical measurement part of the system was almost identical to that used in our previous research^23^. In this study, we used a 485 nm center wavelength diode pulse laser, with an 8 MHz pulse repetition rate, 30 ps full width at half maximum, and 0.8 mW average power. The collimated pulse laser beam was transmitted to a dichroic mirror (MD499; Thorlabs) and divided into two paths: the florescence signal and the IRF signal. A fluorescence signal laser passed through an optical short pass filter (SPF, FF01-498/SP-25; Semrock), and the beam passed through a galvanometer x–y scanner (Cambridge Technologies) consisting of a 4 kHz resonant x-scanner and a 500 Hz non-resonant y-scanner. The scan beam passed through a scan lens, tube lens, mirror, objective lens (20 × /0.45, MPlanFL; Olympus), and image fiber bundle (30,000 core, 600 µm image circle diameter; Fujikura) to illuminate the object. The fluorescence signal from the object passed through the dichroic mirror and filtered using a 500 nm cut-on wavelength optical long pass filter (LPF; cat. no. FFLH0500; Thorlabs). A multimode fiber with a core diameter of 50 μm was used as a pinhole and was connected to a photomultiplier tube (PMT; cat. no. H10720-01; Hamamatsu). The IRF signal was optically delayed using a fiber with a length of 20 m and inputted into the PMT. Because the optical delay method was used, the fluorescence signal and the IRF signal were obtained through the same PMT and the same digitizer channel.

The optical signal acquired through the PMT was converted into an electrical signal and passed through the 10th Gaussian LPF (GLPF, 50 MHz cutoff frequency)^50^ and an electrical amplifier (cat. no. TB-409; Minicircuits). The lowpass signal was acquired using a digitizer (cat. no. PCI-5114; National Instruments) with a sampling rate of 120 MB/s. To ensure sufficient signal quality, the laser signal was averaged over 10,000 times. A digitizer and a GPU (NVIDIA GeForce GTX TITAN Black) implemented in an HP Z420 workstation were used for signal acquisition and data processing.

Trigger synchronization of digitizers, pulsed lasers, and x–y scanners are extremely important to obtain a clear image. Figure 1b shows the signal chronogram of trigger synchronization. Trigger synchronization was implemented using a synchronization block consisting of a D-Flip Flop digital circuit, digital input output, and analog output. First, when an analog signal for operating an x-scan mirror is inputted from the control board to the scan driver, the x-scan driver operates the mirror and yields a 4 kHz synchronization signal. A new trigger is created using the AND operation of the high level of the x-scan synchronization signal and the rising edge of the laser pulse trigger. The generated trigger is used for digitizing triggering and y-scan analog signal generation triggering.

The software was developed in the Microsoft Visual Studio 2013 environment with MFC, NI-Scope API, and Open CV. The GPUs were programmed using NVIDIA’s Common Unified Device Architecture technology^51^. The algorithm processing of individual pixels are independent of one another. Therefore, fluorescence lifetimes for all pixels can be simultaneously extracted^20^.

### Statistical analysis

Data were analyzed by ANOVA using PRISM 9.1.0.221 (GraphPad Software Inc., https://www.graphpad.com/scientific-software/prism/). Statistical significance was accepted for case with p < 0.0001.

### Fluorescence lifetime measurement of fluorescein according to PH

Solutions with different pH were prepared by adjusting the volume of 20% HCl or NaOH in phosphate-buffered saline (PBS). The pH of the solution was measured using a commercially LAQUA 9615S pH meter sold by Horiba. Then, the fluorescence lifetime was measured after adding the same amount of different concentration of fluorescein with 25 μg/mL, 50 μg/mL and 75 μg/mL using excitation wavelength 485 nm and emission wavelength 520 nm by our FLE system or commercialized TCSPC system (FluoTime 300; PicoQuant) at room temperature.

### Cell culture

MDA-MB-361(human breast cancer) and A375 (human melanoma skin cancer) cells were purchased from the Korean cell line bank (Seoul, Korea). MDA-MB-361 and A375 cells were grown in 89% Dulbecco’s modified Eagle’s medium with 10% fetal bovine serum and 1% antibiotic–antimycotic solution. Cells were routinely maintained on plastic tissue culture dishes at 37 °C in a humidified 5% CO2/95% air-containing atmosphere.

### Animal models preparation and In vivo cancer detection

All animal experiments were performed with protocols approved by the Institutional Animal Care and Use Committee (IACUC) of the Asan Institute for Life Sciences, Asan Medical Center. The institutional review board of Asan Medical Center (IRB No.2019-14-272) approved this study. The committee abides by the Institute of Laboratory Animal Resources (ILAR) guidelines. All experiments were performed in accordance with relevant guidelines and regulations. The animals were housed under standard laboratory conditions with a temperature of 21–23 °C and 12 h dark/light cycles. Mice were allowed ad libitum access to food and water throughout the study period. In vivo studies were performed using a xenograft tumor model^52,53^ in mice. In this study, the reproducibility was confirmed by conducting an experiment of 5 animals each according to the type and size of the tumor (n = 5 per tumor size, per tumor type; total n = 20), and representative results were described as a total of 4 animals, one each according to the type and size of the tumor. For the xenograft model, MDA-MB-361 (5 × 106) and A375 (2 × 106) cells were harvested, resuspended in PBS, and subcutaneously injected into the flanks of BALB/c nude mice (6 weeks old; male; body weight, 20 ± 3; Japan, SLC, Inc.). Tumor volumes were monitored using bioluminescent imaging after reaching 60–120 mm3. For in vivo detection of cancer in mice, 0.05 mg/mL sodium fluorescein dissolved in PBS was dropped on the tumor and normal tissues of all mice (n = 20, 5 per tumor size and type). In all cases, in order to completely remove fluorescein remaining on the surface, fluorescein was applied and then the surface was washed with PBS at three times. The animals were administered continuous anesthesia (2% isoflurane) during the measurements (5). Fluorescence lifetime measurements in mouse tissues were performed using an imaging fiber bundle and the developed fluorescence lifetime measurement system. The fluorescence lifetime was measured in real-time by touching the end of the imaging fiber bundle to the site to be measured. When measuring the fluorescence lifetime, to ensure sufficient signal quality, the laser signal was averaged over 10,000 times. The fluorescence lifetime was measured more than once per second. All efforts were made to minimize animal suffering and animal handling was performed in according to the ARRIVE guidelines.

### H&E staining of tissue sections

Tumor tissues in mouse bearing MDA-MB-361 and A375 xenograft models were excised for histological examination. Tissues were dissected, embedded in paraffin, and sectioned (5 μm thick). After then tissues were dewaxed in xylene and rehydrated through a serial of decreasing concentration of ethanol. The tissue sections were washed in PBS and stained with H&E, dehydrated in increasing concentrations of alcohol and xylene, and imaged using a Vectra 3 automated quantitative pathology imaging system.
